# Histone Lysine Methylation and Long Non-Coding RNA: The New Target Players in Skeletal Muscle Cell Regeneration

**DOI:** 10.3389/fcell.2021.759237

**Published:** 2021-12-03

**Authors:** Magdaleena Naemi Mbadhi, Jun-ming Tang, Jing-xuan Zhang

**Affiliations:** Hubei Key Laboratory of Embryonic Stem Cell Research, Department of Physiology, Faculty of Basic Medical Sciences, Hubei University of Medicine, Shiyan, China

**Keywords:** epigenetics, skeletal muscle, skeletal muscle regeneration, histone methylation, lncRNAs, satellite stem cells

## Abstract

Satellite stem cell availability and high regenerative capacity have made them an ideal therapeutic approach for muscular dystrophies and neuromuscular diseases. Adult satellite stem cells remain in a quiescent state and become activated upon muscular injury. A series of molecular mechanisms succeed under the control of epigenetic regulation and various myogenic regulatory transcription factors myogenic regulatory factors, leading to their differentiation into skeletal muscles. The regulation of MRFs via various epigenetic factors, including DNA methylation, histone modification, and non-coding RNA, determine the fate of myogenesis. Furthermore, the development of histone deacetylation inhibitors (HDACi) has shown promising benefits in their use in clinical trials of muscular diseases. However, the complete application of using satellite stem cells in the clinic is still not achieved. While therapeutic advancements in the use of HDACi in clinical trials have emerged, histone methylation modulations and the long non-coding RNA (lncRNA) are still under study. A comprehensive understanding of these other significant epigenetic modulations is still incomplete. This review aims to discuss some of the current studies on these two significant epigenetic modulations, histone methylation and lncRNA, as potential epigenetic targets in skeletal muscle regeneration. Understanding the mechanisms that initiate myoblast differentiation from its proliferative state to generate new muscle fibres will provide valuable information to advance the field of regenerative medicine and stem cell transplant.

## Introduction

Skeletal muscle cells were once understood to have locomotive function only, but advances in medicine and research have shown their essential role beyond just locomotive function. Various studies have documented their crucial role as an endocrine organ that secretes various proteins, like myokines, that regulate energy production and consumption (Iizuka et al., 2014; Giudice and Taylor, 2017). They are also believed to possess anti-tumour protective properties (Stölting et al., 2013). Also, there is a compelling association between various diseases and skeletal muscles, including HIV, neuromuscular diseases, cancer, heart failure, and chronic infectious diseases that induce muscle atrophy, known as cachexia. Muscle loss during cachexia is a malignant condition associated with increased morbidity and mortality (Powers et al., 2016). Finally, skeletal muscles have remarkable regenerative properties that make them especially intriguing to researchers in treating muscular injury and degenerative muscular diseases like Duchenne muscular dystrophy (DMD). Skeletal muscle’s high regenerative capacity is due to their large pool of stem cells called satellite stem cells, which reside beneath the basal lamina of muscle cells (Chang and Rudnicki, 2014). DMD is a genetic disorder caused by mutations in the gene encoding dystrophin characterised by progressive degeneration of muscle cells and weakness. Patridge et al. were the first to explore the restoration of dystrophin using myoblast transplantation in *mdx* mice (Partridge et al., 1989). Their promising results initiated the cascade of clinical trials in muscular dystrophies (Huard et al., 1991; Gussoni et al., 1992; Karpati et al., 1993; Mendell et al., 1995). However, there were still many limitations encountered with the allogeneic transplantation of satellite stem cells. Problems encountered included apoptosis of transplanted myoblast cells, poor migration with intramuscular injection, and host immune rejection. Inoculation with a high number of cells resolved the short-term survival rate and the low migration limitiation (Skuk et al., 1999). However, inoculation with a high number of stem cells increases the body’s stress response which activates a strong immune response and further causes a reduction in the oxygen and nutrition supply of the body (Kuang and Rudnicki, 2008). However, these failing limitations did not discourage the search into exploring more improved therapies in the use of satellite stem cell therapy. Various studies have managed to isolate quiescent satellite cells and successfully differentiate these cells into skeletal muscle cells (Collins et al., 2005; Montarras et al., 2005). Therefore, for satellite stem cell transplantation in regenerative medicine to have a successful clinical application, it is imperative to understand the underlying mechanisms that govern these cells from their quiescent state into fully matured myofiber cells.

The epigenetic control of gene expression is a major regulator of determining satellite stem cell fate via modifying the chromatin structure. This review revises two essential epigenetic regulators, histone methylation and long non-coding RNA (lncRNA) modulation. Histone modification and lncRNA modulation have shown progressive studies in the regulation of myogenesis and appear to be promising potential new targets in degenerative muscle diseases and muscular repair.

### Overview of Skeletal Muscle Development

The initiation of skeletal muscle formation is established by the lineage commitment of satellite stem cells. These satellite stem cells remain quiescent, residing between the basal lamina and sarcolemma of myofibers until they are activated (Mauro, 1961). Upon muscle injury, satellite stem cells are activated and undergo a series of events, including myoblast proliferation, cell migration and differentiation, myotube fusion, and maturation of myofibers (Biressi et al., 2007). These events are regulated by myogenic regulatory transcription factors (MRFs), including MyoD, Myf5, myogenin (MyoG), and MRF4 (Sabourin and Rudnicki, 2000). The various expression markers characterise the different developmental stages of satellite and skeletal muscle cells. During embryogenesis, Pax3 and Pax7 characterise satellite cells, which are derived from embryonic progenitor cells (Relaix et al., 2005; Schienda et al., 2006). In the post-natal life, Pax3 is downregulated, and Pax7 assumes the dominant role of the quiescent adult satellite stem cells (Oustanina et al., 2004; Relaix et al., 2006). After muscle injury, these quiescent adult satellite stem cells dominantly expressing Pax7 are activated, and the cells start to express Myf5, which induces cell proliferation. Deletion of Pax7 resulted in decreased myogenic cells during cell culture due to defects in proliferation (Relaix et al., 2006). Therefore, Pax7 is believed to retain a significant role in the quiescent state of satellite stem cells and the proliferation state. Furthermore, Pax7+/Myf5+ cells are necessary to induce the myogenic marker, MyoD, which withdraws the cell from the cell cycle and initiates myotube cell differentiation (Wilson and Rotwein, 2006). MyoD and Myf5 further have the potential in converting non-myogenic cells, like fibroblast, into muscle cells (Davis et al., 1987; Choi et al., 1990).

MyoD is considered a master regulator of myogenesis for its functional role as an initiator of muscle cell differentiation. MyoD is responsible for activating essential muscle-specific genes of differentiation, such as MyHC, α-actin, troponin isoforms (Sabourin and Rudnicki, 2000), and MyoG (Bergstrom et al., 2002; Beylkin et al., 2006). MyoG initiates myotube fusion (Bergstrom et al., 2002), followed by the maturation of muscle fibre under the control of MRF4 (Megeney and Rudnicki, 1995). The role of MRF4 is not as direct and distinct as the other MRF members and seems to involve a more complex function. MRF4 appears to have a biphasic expression, whereby its expression is activated following the activation of Myf5 during myoblast proliferation and further reactivated during the differentiation phase (Zhu and Miller, 1997; Summerbell et al., 2002; Kassar-Duchossoy et al., 2004). Another study also found that MRF4 and MyoD likely have antagonistic roles during myogenesis (Jin et al., 2007). MRF4 was found to downregulate MyoD to allow for cell proliferation (Jin et al., 2007). However, forced expression of MyoD could inhibit MRF4 and induce partial myoblast differentiation (Jin et al., 2007). In addition, during myoblast differentiation, the overexpression of MRF4 could compensate for myogenin mutant cells (Zhu and Miller, 1997; Sumariwalla and Klein, 2001).

**FIGURE 1:**
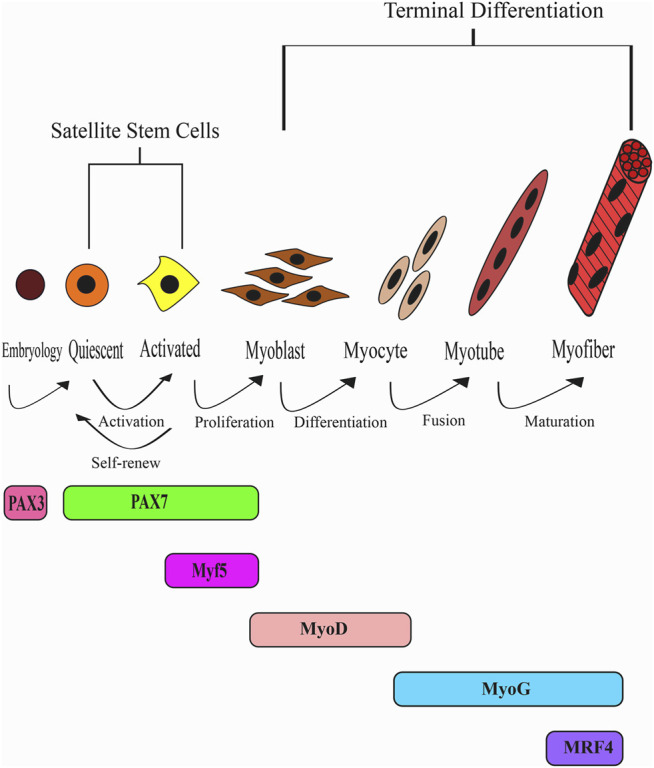
The myogenic regulatory factors (MRFs) pathway during skeletal muscle development and regeneration. Satellite stem cells remain in a quiescent state until activation upon muscle injury. Quiescent satellite stem cells expressing Pax7 are activated to committed progenitor cells expressing Myf5. However, a portion of the cells undergoesself-renewal to regenerate the stem cell pool. Proliferating myoblasts express Myf5 and MyoD while Pax7 is downregulated. MyoD is the master regulator of myoblast differentiation that initiates proliferating myoblast cells to exit the cell cycle. Differentiation and fusion precede to form myofibers under the regulation of MyoG and Mrf4.

Finally, MRFs are believed to function synergistically with the myocyte enhancer factor-2 (MEF2) members to activate myotube formation (Molkentin et al., 1995; Black et al., 1998; Piasecka et al., 2021). Taken together, after muscle injury, Pax7 is downregulated, and the upstream factors, Myf5 and MyoD, determine cell lineage to initiate myogenic differentiation. After that, MyoG and MRF4 expression ensue that function in myotube formation and maturation. Finally, MEF2 synergistically functions together with MRFs to drive myogenesis during muscular regeneration. Figure 1 provides an overview of the development of skeletal muscles from a quiescent state to matured myofibers.

### Histone Modulation During Skeletal Muscle Cell Development

The eukaryotic chromatin is made up of nucleosomes composing of tightly wrapped DNA and histone proteins. Each nucleosome comprises an octamer of histones composed of two molecules of the canonical histone proteins, including H2A, H2B, H3, and H4 and about 150bp of DNA (Luger et al., 1997). The N-terminal histone tails are exposed on the surface of the nucleosome and are susceptible to post-translational modification *via* acetylation, methylation, phosphorylation, and ubiquitylation (Zhang et al., 2015). Post-translational histone modifications regulate the chromatin structure for proper gene regulation and transcription. Transcriptional regulators, including Nanog, Oct4, and Sox2 that maintain the pluripotency of stem cells, must be deactivated for cellular differentiation to occur (Wang et al., 2012; Chu et al., 2016). In addition to transcriptional regulators, induced pluripotent stem cells (iPCS) and embryonic stem cells (ESC) are poised by a bivalent chromatin, consisting of histone 3 lysine 4-trimethylation (H3K4me3) and histone 3 lysine 27-trimethylation (H3K27me3) (Bernstein et al., 2006; Mikkelsen et al., 2007). These bivalent marks are located on the same nucleosome in an asymmetric configuration regulating the chromatin structure (Bernstein et al., 2006). Generally, H3K4me3 is associated with gene activation, while H3K9me3 and H3K27me3 are associated with gene repression (Black et al., 2012). Upon receivingdifferentiation cues, the H3K27me3 repressive mark is lost, and H3K4me3 activity dominates the promoter region lineage-specific genes to activate gene transcription (Collinson et al., 2016). The bivalent gene is essential to regulate the transition between pluripotency and committed cells. Moreover, the H3K27me3 repressive mark of the bivalent gene maintains low expression levels of developmental genes in iPCS and ESC while allowing for their transcription upon differentiation. Furthermore, H3K27me3 protects the cell from aberrant gene activation by permitting only the target lineage-regulating genes to activate (Vastenhouw and Schier, 2012). H3K4me3 has vital significanceas a transcriptional activator. In addition to this main function, H3K4me3 may be essential to ensure that permanent gene silencing of developmental genes does not occur (Fouse et al., 2008; Vastenhouw and Schier, 2012).

During their quiescent state, satellite stem cells possess a similar bivalent chromatin (Pan et al., 2007; Liu et al., 2013; Akiyama et al., 2017). Interestingly, using ChiP-seq analysis, Liu et al. (Liu et al., 2013) found that the large number of genes containing H3K27me3 in quiescent satellite cells were non-myogenic genes and that the myogenic genes lacked H3K27me3. Furthermore, the knockout of the polycomb repressor complex 2 (PRC2)-Ezh2, responsible for the deposition of H3K27me3, in myogenic progenitors resulted in the activation of the non-myogenic gene lineages (Juan et al., 2011). This observation further highlights the relevance of H3K27me3 of the bivalent chromatin to suppress the activation of alternative lineage regulators, thus regulating cell fate.

For differentiation of skeletal muscle cells to commence, three requirements need to be met: 1) Loss of repressive marks on the chromatin; 2) Activation of the permissive marks to promote gene expression; 3) Promotion of RNA PolII on the promoter of the chromatin.

Upon activation of satellite stem cells after muscle injury, they undergo one of the two fates, symmetrical cell division or asymmetric cell division. In symmetric cell division, satellite cells self-renew by giving rise to identical cells that re-enter the cell cycle to maintain the quiescent pool of satellite stem cells. Contrarily, asymmetrical cell division results in satellite stem cells giving rise to two daughter cells, one identical satellite stem cell and one committed cell that will undergo proliferation (Kuang et al., 2007). Demethylation of H3K27me3 is mediated by the Jummji C (JmjC) domain-containing demethylases UTX andJMJD3 (Agger et al., 2007; Shpargel et al., 2014). Interestingly, proceeding studies found that it is mainly the upregulation of JMJD3 and not UTX that results in H3K27me3 demethylation to drive the differentiation into committed myogenic cells (Akiyama et al., 2016). Upon the loss of H3K27me3, H3K4me3 activity is associated with euchromatin and promotes RNA polymerase II to the chromatin (Pekowska et al., 2011). This permits a less compacted chromatin structure that allows for gene transcriptional expression. These underlying mechanisms are discussed below:

## Histone Modification in Muscle Development and Regeneration

### H3K27 Modulation

The bivalent gene consisting of H3K27me3 and H3K4me3 (Bernstein et al., 2006) is associated with the heterochromatin because the H3K27me3 repressive mark in developmental genes maintains dominance. H3K27me3 is present in myoblasts and myotubes and regulates differentiation by silencing muscle-specific genes (Asp et al., 2011), including MyoD and MyoG (Dilworth and Blais, 2011). Upon satellite stem cell activation and lineage commitment, H3K27me3 silences differentiation muscle-specific genes, thus allowing cell proliferation. The Polycomb group proteins (PcG) consisting of the KMT6 family is responsible for depositing H3K27me3 on target promoter regions. The PcG protein Ezh2 is a subunit of the polycomb repressor complex 2 (PRC2) recruited on the promoter of regulatory genes and catalyses H3K27me3 resulting in gene inactivation (Palacios et al., 2010). The mechanism involves the phosphorylation of Ezh2 by MAPK signalling (p38), which permits YY1 to recruit the phosphorylated Ezh2 to promoter regions (Palacios et al., 2010). It is further believed that additional factors, such as the histone demethylase Jarid2, are also required to recruit Ezh2 to the promoter of target genes (Peng et al., 2009; Pasini et al., 2010).

Muscle cell differentiation requires the loss of H3K27me3 from promoter regions of differentiation regulating genes like MyoG. Various mechanisms have been identified in this process. The KDM6 family member, UTX, was reported to function in a two-step mechanism to demethylate H3K27me3 at target muscle genes (Seenundun et al., 2010). Firstly, the homeobox protein Six4 recruits UTX to regulatory regions of target genes, including MyoG, and the demethylation of H3K27me3 occurs at localised regions. Upon UTX association with a specific locus, H3K27me3 demethylation activity requires the elongation of RNA PolII to spread across the genome (Seenundun et al., 2010). Interestingly, this study also found that inhibition of the RNA Pol II elongation resulted in establishing the bivalent H3K27me3/H3K4me3 gene with increased Ezh2 and Suz12 expression and loss of UTX within the coding regions. The inhibition further resulted in the loss of MyoG expression.

Msk1 kinase has also been found to play a significant role in eliminating H3K27me3 from muscle-specific genes via the phosphorylation of histone three serine 28 (H3S28) (Stojic et al., 2011). This association depreciates Ezh2 interaction on promoter regions of target genes and instead favours binding of Ezh1, which possess weaker H3K27me3 activity (Margueron et al., 2008). Furthermore, the presence of Ezh1 is required for the recruitment of RNA Pol II to MyoG for transcription (Mousavi et al., 2012). Finally, UTX is then introduced to remove H3K27me3, including demethylating the weak marks established by Ezh1, thus ensuring complete loss of the repressive mark on the chromatin of muscle for gene activation (Seenundun et al., 2010).

The incorporation of histone variants H3.3 into differentiation-specific genes is necessary for gene activation (Harada et al., 2012). Furthermore, H3.3 and H3.1 appear to possess antagonistic effects on the state of H3K27me3 with knockdown of H3.3 decreasing H3K27me3 while expression of H3.1 increased H3K27me3 inhibiting myoblast differention (Harada et al., 2015). MEF2 causes a shift from H3.1 to H3.3 *via* the histone chaperon HIRA, thus eliminating H3K27me3 repression for gene activation (Yang et al., 2011).

Once the H3K27me3 repressive mark is removed, activation of TrxG complex (Ash2L) via Mef2d and Six1 is then allowed, which deposits trimethylation on H3K4, resulting in a euchromatin structure (Rampalli et al., 2007).

### H3K9 Modulation

The Suv391H1 methyltransferase catalyses the di- and tri-methylation of H3K9 (Rea et al., 2000; Fritsch et al., 2010). Suv391H1 is recruited via interaction with MyoD to the promoter regions of muscle target genes resulting in H3K9me3 and gene repression (Mal and Harter, 2003). The mechanisms that modulate Suv391H1-MyoD interaction involves the phosphorylation of MyoD by p38γ MAPK (Mal, 2006). Various studies have confirmed that during the proliferation phase of myoblast cells, MyoD recruits H3K9me2 and H3K9me3 repressive marks on the promoter region of MyoG, hence, inhibiting myoblast differention (Zhang et al., 2002; Mal and Harter, 2003). Furthermore, in C2C12 cell lines, overexpressing Suv391H1 continues to inhibit differentiation-specific genes even in differentiation-inducing media (Mal, 2006). Deposition of H3K9me3 by Suv39h1 appear to repress the early muscle genes (Mal, 2006), while deposition of H3K27me3 by Ezh2 functions on repressing the late genes of undifferentiated cells (Caretti et al., 2004). In addition to maintaining myoblast cells in the proliferative phase, Suv39h1 also has a functional role in mediating muscle-specific genes involved during terminal differentiation (Ait-Si-Ali et al., 2004).

G9a is a member of the SET domain-containing Suv39 family (Tachibana et al., 2001) that catalyses the methylation of H3K9 (Tachibana et al., 2002; Yokochi et al., 2009; Ling et al., 2012). During myoblast proliferation, G9a deposits H3K9me2 on MyoD, repressing its transcriptional activity (Ling et al., 2012; Wang and Abate-Shen, 2012). This is achieved via a homeodomain repressor, Msk1, interaction with G9a on the Myod1 locus (Lee et al., 2004). Interaction of Msk1 with G9a leads to the deposition of the H3K9me2 repressive mark and inhibition of differention (Wang and Abate-Shen, 2012). However, the extensive function of G9a in skeletal muscles is still under investigation as one study reported on its redundant function, observing no phenotypic change after G9a knockout in mice skeletal muscles (Zhang et al., 2016).

Choi et al. (Choi et al., 2010) were the first group to report on the demethylation mechanisms of histones in myogenesis. MEF2 recruits the histone demethylase LSD1 that removes the H3K9me2 and H3K9me3 repressive marks from promoter regions of muscle-specific genes to promote muscle cell differentiation (Choi et al., 2010). Studies found that inhibition of LSD1 maintained the H3K9me2 repressive mark on the promoter regions of MyoG and MCK, inhibiting myoblast differentiation (Choi et al., 2010). JMJD2A (Kdm4a) is also required to remove the Suv39h1 mediated H3K9 methylation, and it is via JMJD2A mechanisms that LSD1 appears to be facilitated (Verrier et al., 2011). KDM4B is another H3K9 demethylation enzyme that acts on the promoter region of MyoD. However, unlike LSD1, which is essential for myogenic differentiation, KDM4B appears only to enhance myogenic differentiation as its depletion did not completely block myogenic differentiation but instead delayed it (Choi et al., 2015). Interestingly, H3K9 and H3K4 methylation appear to be mutually exclusive (Wang et al., 2001). Thus, to maintain the demethylation of H3K9, the Set7/9 methyltransferase enzymes must deposit H3K4me1 on MyoG to ensure that H3K9me3 is not re-introduced (Tao et al., 2011).

### H3K4 Modulation

Myoblast differentiation requires the loss of repressive marks and the addition of permissive marks at promoter regions of muscle genes. There are four well-established permissive markers of myogenic differentiation, and these include H3K4me1, H3K27ac, p300, and RNA II polymerase (Blum et al., 2012). The active enhancer H3K4me1 deposition is regulated by H3-H4 histone methyltransferase Set7 (Tao et al., 2011). Studies found that Set7 directly interacts with Myod to regulate gene expression (Tao et al., 2011). As previously mentioned in the last section, the depletion of the H3K9me3 repressive mark appears to be mutually exclusive with the introduction of H3K4me1 by Set7/9^87^. Indeed, inhibition of Set7 resulted in decreased H3K4me1 levels and MEF2 expression, impairing skeletal muscle differentiation (Tao et al., 2011). Furthermore, the inhibition of Set7 resulted in decreased levels of H3K4me1 with a loss of MyoD, MyHC, and MyoG expression (Wang et al., 2001; Nishioka et al., 2002; Tao et al., 2011). This ultimately resulted in a decreased number of myotube formation and impaired skeletal muscle differentiation (Tao et al., 2011).

At active promoters, H3K4me1 flanks with H3K4me3 in a bimodal pattern (Bae and Lesch, 2020). The H3K4me3 permissive mark in satellite stem cells is established via TrxG complexes (Ash2L/MLL2 methyltransferases) (McKinnell et al., 2008; Diao et al., 2012; Kawabe et al., 2012). The transcriptional factor Pax7 mediates the recruitment of Ash2L/MLL2 through binding upstream of the Myf5 transcriptional start site (TSS) (McKinnell et al., 2008; Soleimani et al., 2012). The methylation of arginine residues on the amino-terminus of Pax7 by arginine methyltransferase CARM1 is another factor that recruits TrxG complexes (McKinnell et al., 2008; Kawabe et al., 2012). By this methylation of Pax7 by CARM1, Pax7 can interact with Ash2L/MLL2 to target the Myf5 promoter region to deposit H3K4me3 and activate gene expression for myoblast proliferation (McKinnell et al., 2008; Diao et al., 2012; Kawabe et al., 2012). Similarly, Ash2L/MLL2 also targets the MyoG promoter region to deposit H3K4me3 and initiate proliferating myoblasts to commit to differentiation (Rampalli et al., 2007). Ash2L/MLL2 is recruited to the promoter region of MyoG via interactions with phosphorylated MEF2D by p38-α (Rampalli et al., 2007).

Furthermore, studies found that H3K4me3 co-localizes with the KDM5B demethylase enzyme at promoter regions of active genes (Kidder et al., 2014; Xue et al., 2020). Inhibition of KDM5B resulted in widespread H3K4 methylation into gene bodies, leading to defects in gene expression and impairment in the self-renewal and differentiation of embryonic cells (Kidder et al., 2014). Thus, it is proposed that KDM5B has significance in localising H3K4 methylation at the promoter and enhancer regions of relevant genes (Kidder et al., 2014; Xue et al., 2020). Whether similar regulatory mechanisms of KDM5B in muscle-regulated genes also exist is yet to be explored.

PARP1, a member of the Poly (ADP-ribose) polymerases family, has also been found to coordinate MyoD gene expression by regulating H3K4me3 (Matteini et al., 2020). In skeletal muscle cells, PARP1 impairs the accumulation of the permissive marker, H3K4me3, on the MyoD binding site, which inhibits myogenic gene expression. It is postulated that PARP1 directly interacts with KvDMR1 (Matteini et al., 2020), an imprinting control region necessary for MyoD regulation of muscle cell differentition (Busanello et al., 2012; Andresini et al., 2016). Interestingly, PARP1 appears to possess a cell-specific function in the regulation of H3K4me3. In its active state, PARP1 inhibits the H3K4me3 demethylase enzyme, KDM5B, and increases H3K4me3 in MCF-7 cells (Krishnakumar and Kraus, 2010). In contrast, in the HEK293T cell lines, the inactive state of PARP1 inhibited H3K4me3 in IL-6 by hindering methyltransferase MLL (Minotti et al., 2015).

### Other Histone-Methyl Modulation

SUV4-20H1 is a dimethyltransferase that mediates the deposition of H4K20me3 (Boonsanay et al., 2016). Deposition of H4K20me2/3 has significance in maintaining satellite stem cell quiescent state (Boonsanay et al., 2016). One study demonstrated that SUV4-20H1 appears to deposit H4K20me2 on the MyoD locus and thus, inhibiting myoblast differentiation (Boonsanay et al., 2016). Inhibiting SUV4-20H1 decreases H4K20me2 expression on the MyoD distal regulatory region (DRR) and increases the permissive mark H3K4me3 (Boonsanay et al., 2016). This resulted in the activation of muscle stem cells with significant depletion of the quiescent stem cells impairing muscle cell regeneration capacity after muscular injury. Interestingly, the deletion of SUV4-20H1 led to the decreased expression levels of H3K20me2 and significantly decreased H3K27me3 levels (Boonsanay et al., 2016).

The SET domain containing 2 (Setd2) is an H3K36me3 that modulates the chromatin to an active state (Kouzarides, 2007; Edmunds et al., 2008). It was found that inhibition of Setd2 in the C2C12 cell line resulted in aberration in myotube formation with decreased expression of MyHC and MyoG (Yi et al., 2017). Interestingly, silencing of Setd2 did not appear to affect MyoD, Myf5, and MRF4 (Yi et al., 2017). Furthermore, silencing of Setd2 arrested the cell cycle and upregulated cyclin-dependent kinase inhibitor p21 resulting in decreased proliferation rate (Yi et al., 2017). The mechanisms involved are yet to be identified.

**FIGURE 2:**
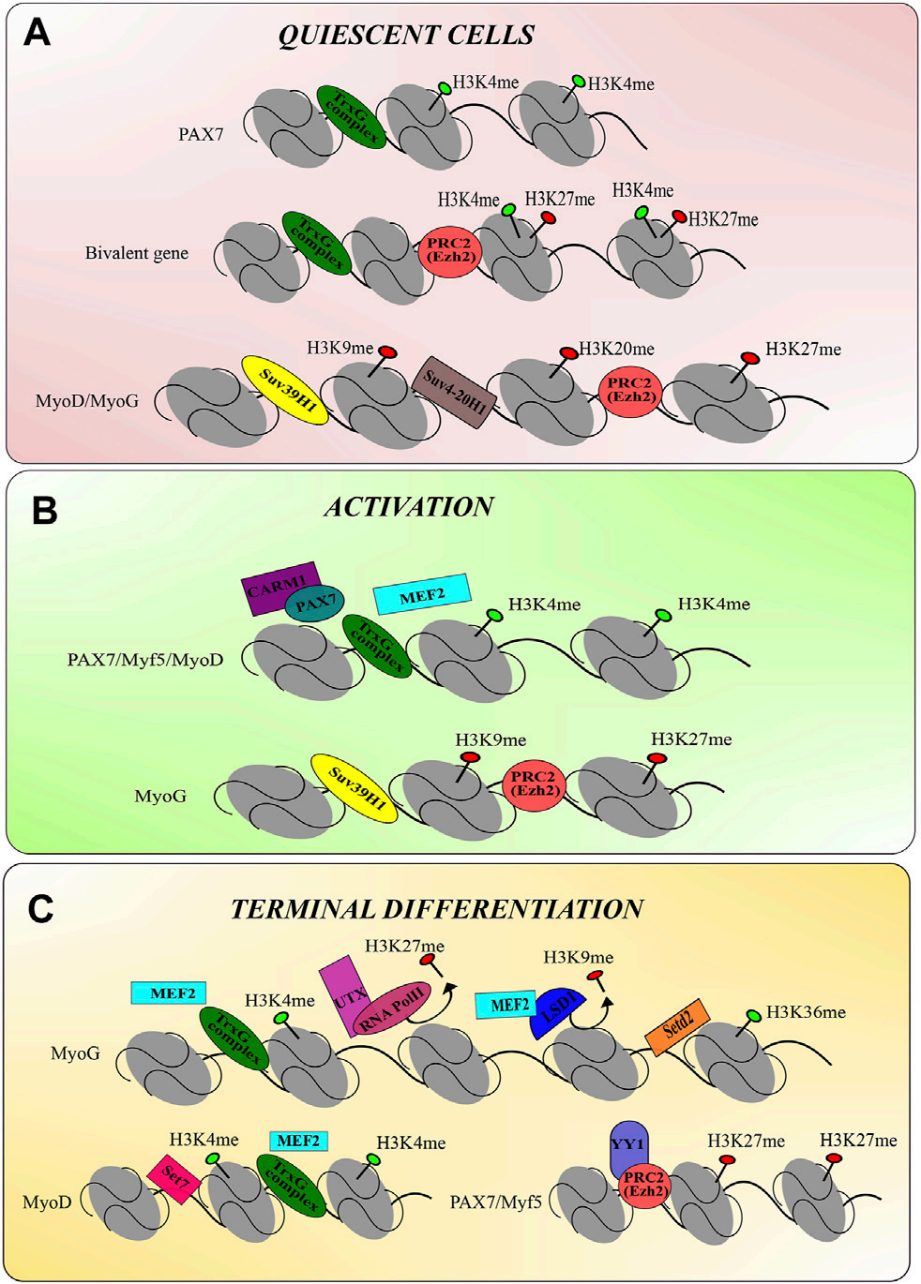
The epigenetic modulation of histone methyltransferases during skeletal muscle regeneration. **(A)** Epigenetic mechanisms of histone methyltransferases in quiescent stem cells. TrxG-mediated H3K4me catalyses the permissive chromatin of Pax7. MyoD and MyoG gene expression is repressed by H3K27me, H3K9me, and H3K20me via their respective enzymes. Interestingly, the bivalent gene consists of an activating H3K4me mark and the repressive H3K27me, however, H3K27me activity dominates to repress gene transcription during the quiescent state. **(B)** Epigenetic mechanisms of histone methyltransferases in activating progenitor cells. Upon the activation of stem cells, the permissive chromatin of Pax7, Myf5 and MyoD is catalysed by deposition of H3K4me via TrxG complexes. MEF2 and Carm-Pax7 complex recruits TrxG complexes to establish the permissive chromatin. MyoG remains with the repressive chromatin marks, H3K27me and H3K9me, catalysed by PRC2 and SUV39H1, respectively. **(C)** Epigenetic mechanisms of histone methyltransferases in during terminal differentiation. The MyoG chromatin permits gene transcription via H3K4me and H3K36me marks by TrxG complexes and Setd2, respectively. UTX and RNA PolII remove the repressive mark H3K27me, and MEF2 recruits LSD1 to remove the H3K9me repressive mark. MyoD gene transcription continues via the activating H3K4me. Pax7 and Myf5 are silenced by recruitment of PRC2 by YY1, which deposits the H3K27me repressive mark.

As the continual exploration into new epigenetic regulatory mechanisms is underway, these observations demonstrate the significant role that histone modifications play in muscle cell development and regeneration. From maintaining the quiescent stem cell pool through muscle cell activation and maturation of muscle cells, histone modifications may provide new novel target therapy in muscle dystrophies. Figure 2 provides a comprehensive summary of the current theories of histone methyltransferase modulations during skeletal muscle development.

### LncRNA in Myogenesis

Long non-coding-RNA (lncRNA) are a class of non-coding RNA molecules with more than 200 nucloetides (Engreitz et al., 2016). In epigenetics, lncRNAs have been reported with various functions. This includes: 1) mediating chromatin remodelling and modification (McHugh et al., 2015); 2) interacting with transcriptional factors for gene regulation (Atianand et al., 2016); 3) interacting with mRNAs to regulate the post-transcriptional processes of mRNAs (Tripathi et al., 2010; Wang et al., 2016). Recent studies have found that lncRNAs have significant roles in coordinating muscle-specific transcriptional factors during the different phases of myogenesis. LncRNAs direct epigenetic regulators to mediate MRFs during muscle cell development and regeneration (Dey et al., 2014; Wang et al., 2016; Chen et al., 2017; Xu et al., 2017). Furthermore, lncRNAs coordinate with muscle cell regeneration regulatory factors after contusion injury to regenerate muscle cells (Zheng et al., 2019). Amongst their diverse functions with other molecular factors, they also possess a cell-type-specific expression characteristic that makes them vital cell determination and commitment (Cesana et al., 2011; Wang et al., 2013). Below, we present lncRNAs that have been of recent interest in muscle cell development and regeneration.

### H19

LncRNA H19 was one of the first lncRNAs identified with a crucial role in skeletal muscle. It promotes muscle cell differentiation and functions in skeletal muscle regeneration after injury (Dey et al., 2014). The mechanisms by which H19 works to promote skeletal muscle regeneration have been identified to involve inhibiting the myoblast inhibitory genes, Sirt1 and FoxO1, that leads to increased expression of MyoG and MyHC (Xu et al., 2017). Also, H19 is reported to encode two microRNAs (miRNA), miR-675-3p and miR-675-5p, which promote skeletal muscle cell differentiation (Dey et al., 2014). MiR-675-3p and miR-675-5p target the anti-differentiation factors, Smad proteins and Cdc6, respectively (Dey et al., 2014). MiR-675-3p and miR-675-5p are able to rescue abnormal muscle regeneration in H19 knockout (Dey et al., 2014). The significance of H19 during skeletal muscle regeneration was also evident after skeletal muscle injury in mice, where its expression was significantly increased during the early phase of muscle repair (Zheng et al., 2019). Further, the increase in H19 expression positively correlated with Myf5, MyoG and the angiogenic factors, HIF-1α and Angpt1 (Zheng et al., 2019). Interestingly, one study presented the discovery of a negative-feedback loop between H19-Igf2-MyoD (Borensztein et al., 2013). In this study, the knockdown of MyoD and Igf2 in mice models affected the development of muscles of the diaphragm (Borensztein et al., 2013). They reported that MyoD binds to the CS9 mesodermal enhancer of H19, thereby interacting with the promoter region of H19. This interaction increases the expression of H19, which results in trans repression of Igf2 (Wilkin et al., 2000). The repression of Igf2 further results in the negative regulation of MyoD via the suppression of the SRF transcription factor (Borensztein et al., 2013). Thus, H19 is essential for the development of muscle cells via regulation of key muscle development factors and pathways, including, Sirt1, FoxO1, microRNAs, and Igf2.

### LncMyoD

During skeletal muscle differentiation, MyoD activates lncMyoD (Gong et al., 2015) and lnc-YY1 (Zhou et al., 2015) and promote skeletal muscle differentiation. Increased expression of LncMyoD is observed during muscle regeneration and development, and its knockdown negatively regulates a key number of muscle-related mRNAs (Gong et al., 2015). The mechanisms involve the binding of lncMyoD to IGF2-mRNA-binding protein 2 (IMP2), which negatively regulates the proliferation genes, N-Ras and c-Myc (Gong et al., 2015). The negative regulation of these genes then permits exit of the cell-cycle, thus, promoting myoblast differentiation (Gong et al., 2015). In addition to promoting myoblast differentiation, lncMyoD further has a regulatory role in the composition of muscle fibre-type (Zhang et al., 2021). Like most lncRNAs, lncMyoD also has the property of sponging miRNAs to activate transcription. Via the sponging of miR-370-3p, lncMyoD can regulate muscle fibre type composition (Zhang et al., 2021). LncMyoD promotes differentiation with an increased enhancement of fast muscle fibre while decreasing slow muscle fibre (Zhang et al., 2021). Correspondingly, *in vitro* studies in C2C12 cell lines revealed that the inhibition of lncMyoD upregulated miR-370-3p expression inhibiting myoblast differentiation, increasing slow muscle fibre formation, and decreasing fast muscle fibre formation. Furthermore, lncMyoD could rescue the inhibitory effects of miR-370-3p to promote myotube differentiation and switch to the formation of fast muscle fibres (Zhang et al., 2021). Conclusively, lncMyoD inhibits myoblast proliferation while promoting differentiation and regulates muscle fibre composition.

### LncYY1

Lnc-YY1 is activated by MyoD and possess regulatory functions to activate myotube differentiation. Upon the activation of MyoD, lncYY1 destabilises the YY1/PRC2 repressive complex from the promoter region of target genes, which frees the chromatin and allows for gene activation (Zhou et al., 2015). During muscle regeneration, inhibition of lncYY1 decreased expression of Pax7, MyoD, MyoG, and MyHC (Zhou et al., 2015). Furthermore, together with lncRNA H19, lncYY1 shows a more prominent role during the early phase of skeletal muscle regeneration (Zheng et al., 2019). The expression pattern of lncYY1 during myogenesis display low expression in proliferating myotubes, peak expression during the early phase of myotube differentiation, and decreased expression during the late differentiation phase (Zhou et al., 2015). Studies found that inhibition of H19 or lncYY resulted in decreased expression of MyoD and MyoG, leading to the aberration of skeletal muscle repair post-injury (Cesana et al., 2011; Dey et al., 2014; Zhou et al., 2015). Therefore, lncYY1 may regulate significant key pathways necessary for myotube differentiation via MyoD and MyoG-related mechanisms.

### Malat1

Metastasis-associated lung adenocarcinoma transcript 1 (Malat1) was initially identified in non-small cell lung cancer (NSCLC) as a prognostic marker (Ji et al., 2003). Watts et al. (Watts et al., 2013) were the first to report on the role of Malat1 in myogenesis. They found that Malat1 expression was upregulated during myoblast differentiation. Silencing of Malat1 resulted in decreased expression of MyoG and decreased myotube differentiation. The *in vivo* and *in vitro* studies further revealed that Malat1 was suppressed by myostatin, a negative regulator of myogenesis (Watts et al., 2013). Similarly to most lncRNAs, Malat1 functions as a sponge for miRNAs in the regulation of myogenesis. Malat1 consists of a miR-133 target region that targets serum response factor (SRF) to promote myoblast differentiation (Han et al., 2015). The inhibition of Malat1 resulted in decreased expression of SRF and inhibited differentiation during *in vitro* studies of C2C12 cells (Han et al., 2015). Further, inhibition of Malat1 arrested the cell-cycle in G0/G1 phase affecting myoblast proliferation and decreased MyoG expression levels (Watts et al., 2013). However, a contrasting study observed Malat1 acting as a repressor of myogenesis (Chen et al., 2017). In this study, Malat1 recruited Suv391h1 on the Myod1 loci during myotube proliferation. Differentiation cues degraded Malat1, destabilising the Suv391ha-complex resulting in the activation of MyoD. Furthermore, inhibition of Malat1 during *in vivo* studies of the mdx mouse model of muscular dystrophy enhanced muscle regeneration capacity, increasing muscle fibres (Chen et al., 2017). Similarly, during skeletal muscle repair after contusion injury, there was a parallel interaction between Malat1 expression with MyoD and MyoG (Zheng et al., 2019). These contrasting observations further make Malat1 an intriguing investigation because even with its abundant expression in all human tissues, its knockdown does not exhibit any obvious phenotypical aberration during development (Eißmann et al., 2012; Nakagawa et al., 2012; Zhang et al., 2012). Beyond myotube formation, Malat1 is further implicated in regulating the inflammatory response during skeletal muscle repair post-injury (Marques-Rocha et al., 2015; Zhao et al., 2016; Huang et al., 2017). There are indeed many inflammatory cytokines, including TNF-α, IL-6, and IL-10, that were found to positively correlate with Malat1 post-muscular injury (Zheng et al., 2019). Malat1 appears to regulate inflammatory activities via the recruitment of EZH2 (Yong et al., 2020). During sepsis of muscle tissue, Malat1 interacted with EZH2 to increase inflammatory cells and increase muscle cell apoptosis. In summary, Malat1 appears to have inconclusive yet a regulatory role during myogenesis with significant influence during the inflammatory response of skeletal muscle repair.

### Myparr

The lncRNA Myparr is transcribed by the MyoG promoter region (Hitachi et al., 2019). Myparr functions to regulate myogenesis by two mechanisms. Firstly, Myparr activates the myogenic regulatory miRNAs to withdraw the myoblast cell from the cell-cycle and initiate myoblast differentiation (Hitachi et al., 2019). Secondly, Myparr regulates MyoG to promote myoblast differentiation (Hitachi et al., 2019). Studies found that silencing of Myparr resulted in decreased occupancy of PolII and a significant reduction in the permissive marks, H3K4me3 and H3K27ac, on the MyoG locus. The underlying mechanisms involve regulating the interaction of Ddx17, a coactivator of MyoD, with PCAF proteins (Hitachi et al., 2019). Interestingly, there also appears to exist a positive-feedback between Myparr and MRFs. During the inhibition of MyoG, compensatory mechanisms enhanced the expression of Myparr, together with Myf5 and MRF4 (Hitachi et al., 2019).

**FIGURE 3:**
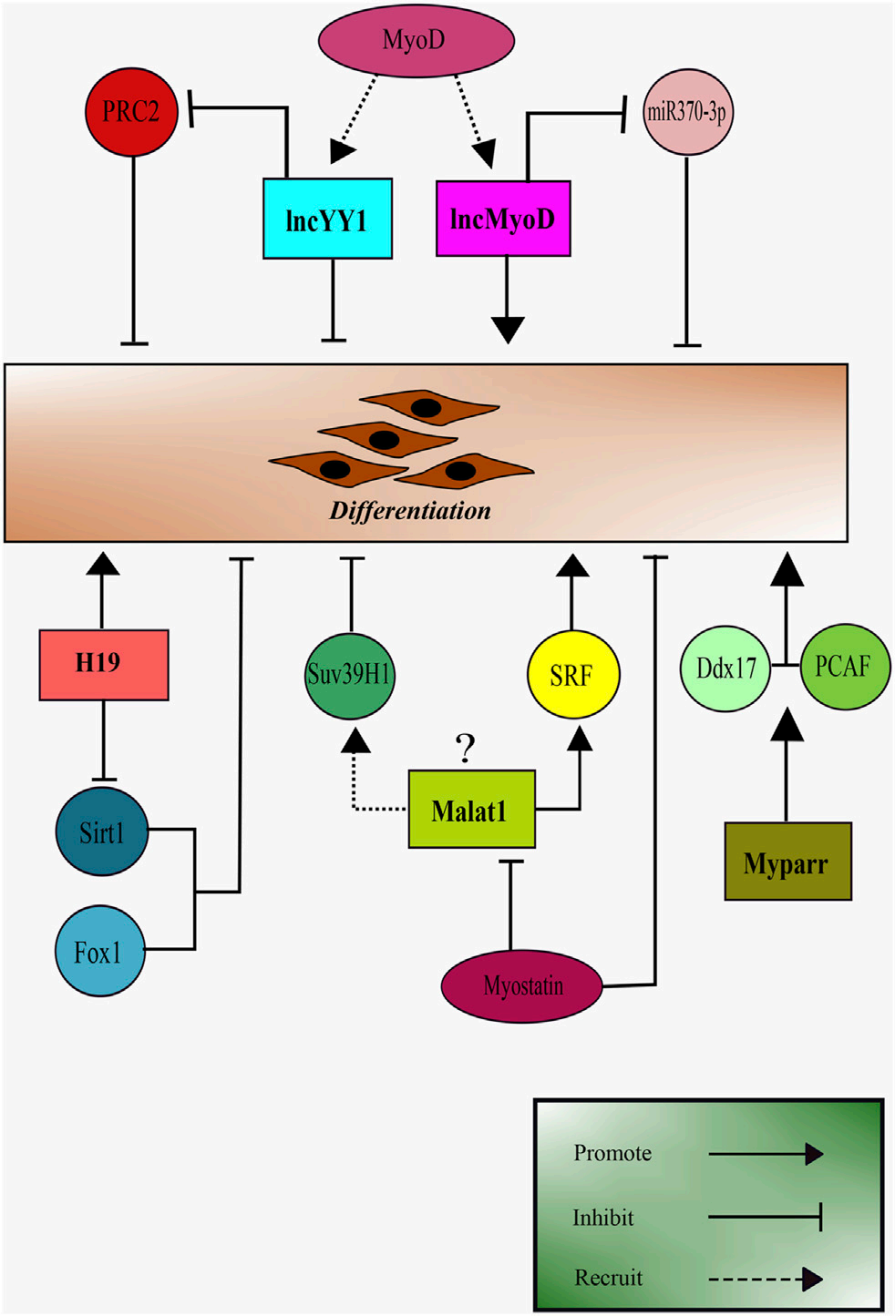
Schematic representation of LncRNAs role in skeletal muscle differentiation. LncRNA H19, lncMyoD, lncYY1, and Myparr promote myoblast differentiation. Malat1 appears to have contrasting results being a promoter of differentiation and recruiting SUV39H1, an inhibitor of differentiation.

As summarised in Figure 3, there are several emerging evidence of the role of lncRNAs during myoblast differentiation into matured muscle cells. LncRNAs possesses great potential as effective targets during muscular regeneration. It is worth exploring further for their upstream regulations during myogenesis for a comprehensive understanding of their potential in clinical application.

### Long Non-coding RNAs Interaction With Histone Methylation Proteins

More than just their interaction with MRFs, lncRNAs further have underlying mechanisms with epigenetic proteins, including histone methylation enzymes to regulate gene expression. Their functional roles include recruitment of histone methylation proteins and acting further as a molecular scaffold to coordinate the localisation of these epigenetic complexes (Hanly and Esteller, 2018). One of the well-documented roles of lncRNAs regulation of epigenetic modulators is their mediation of gene suppression via interaction with PRC2 (Hanly and Esteller, 2018). LncRNAs function as a molecular scaffold to coordinate the localisation and binding of PRC2 on promoter regions of target genes to deposit H3K27me3 (Rinn et al., 2007; Tsai et al., 2010; Yap et al., 2010). Furthermore, an individual lncRNA has the unique ability to harness different histone enzymes on their distinct domains. Tsai et al. (Tsai et al., 2010) found that while the 5′-end of lncRNA HOTAIR binds with the PRC2 complex, its 3′-end binds with the H3K4 histone demethylase, LSD1, to coordinate gene repression. LncRNAs were also found to recruit G9a, responsible for H3K9me3 deposition, to silence target genes in mouse placental tissue (Nagano et al., 2008) and to coordinate lineage-specific transcriptional silencing of target genes (Pandey et al., 2008). Whether the aim is to turn genes off via H3K27me3, H3K9me3, or demethylate H3K4me3, there is expanding evidence of the significant role of lncRNAs coordination gene suppressive effects. An interesting observation about the target mechanisms of lncRNAs appears to be highly cell type-specific. In relation to cancer cells, lncRNA ANRIL mediates the repression of tumour suppressor proteins via the recruitment of PRC1 and PRC2 (Aguilo et al., 2011). However, in smooth muscle cells, the levels of H3K27me3 were unaffected by the inhibition of ANRIL during the phenotypic switching of aortic smooth muscle cells (Zhang et al., 2020). Instead, ANRIL acted as a molecular scaffold for the WD-40 repeat-containing protein 5 (WDR5) and histone deacetylase HDAC3 complex to activate gene transcription *via* H3K4me3 (Zhang et al., 2020). In developmental genes, lncRNA HOTTIP is present at the bivalent gene, and its transcription resulted in increased H3K4me3 levels and decreased H3K27me3 (Wang et al., 2011). Similarly toANRIL, HOTTIP mediated gene activation by modulating the localisation and occupancy of WDR5 to deposit H3K4me3 (Wang et al., 2011). Furthermore, the loss of HOTTIP led to the diffusespread of MLL and WDR5 across the gene body and decreased H3K4me3 levels at the promoter region of the HOXA gene (Wang et al., 2011). These studies provide compelling evidence of the specificity of lncRNAs to modulate epigenetic events via the methylation of histone proteins. Thus, these discoveries about lncRNAs make them particularly intriguing in stem cell therapy to mediate epigenetic events during cellular differentiation.

### Epigenetic Therapy in Muscle Diseases and Muscle Regeneration

The evidence above provides a strong indicator that epigenetic modulations like histone methylation and lncRNA alter gene expression. The dysregulation of any of these processes can lead to aberrations in muscular development and regeneration. Therefore, a comprehensive understanding of these epigenetic changes can advance the development of epigenetic drugs to treat muscular dystrophy diseases and enhance satellite stem cell therapy. Thus far, histone deacetylase inhibitors (HDACi) are the only epigenetic drugs that have been well studied and used in clinical trials to treat muscular dystrophies. HDACi inhibits myostatin activity, resulting in increased formation of myotubes to treat DMD (Myostatin, 2008). Givinostat is the first HDACi used in the treatment of muscle diseases. It showed a successful increase in myofiber mass and size in mdx mice (Minetti et al., 2006) and showed increased muscle tissue accompanied with decreased fibrotic tissue and necrosis in clinical trials of DMD patients (Bettica et al., 2016). However, a major challenge of using epigenetic drugs is their non-enzyme specificity effects. The coordination between methylation substrates and acetylation of several histone lysines often results in off-target enzyme activation with the use of HDACi (Huang et al., 2011; Zentner and Henikoff, 2013). Therefore, as successful as these studies are, HDACi has systematic effects that influence many gene expressions. Thus, they are often accompanied by major side effects, including nausea, neutropenia, thrombocytopenia, or ventricular arrhythmia (Suraweera et al., 2018). In contrast, lncRNAs function to regulate gene expression in a locus and allele-specific way. They appear to function in a more tissue-type specific manner (Parasramka et al., 2016).

Because of these various histone cross-talks, exploring combinatorial drug therapies between HDACi and histone methyltransferase drugs may augment therapeutic effects than serving an individual drug target. Indeed, there is a growing interest in the innovation of methyltransferase inhibitors in the clinic. EZH2 is a catalytic enzyme for polycomb repressive complex 2 (PRC2) responsible for the repressive H2K27me3 mark (Marchesi et al., 2014). At the initiation of skeletal muscle differentiation, EZH2 is displaced from promoter regions of muscle-specific genes, and KDM6a, a histone demethylase enzyme, is recruited (Seenundun et al., 2010). Further, the overexpression of EZH2 leads to impaired muscle differentiation (Caretti et al., 2004). It is thus essential to destabilise this component from target genes for myogenesis to occur. EZH2 inhibitor drugs demonstrate a high potency against EZH2 and are highly selective (Morera et al., 2016). They also exhibit tolerable side effects, including nausea, asthenia, anorexia, dyspnea, and anorexia that were of low grade (Knutson et al., 2014). Synthetic histone demethylase-mimicking drugs are also a great potential for therapeutic exploration.

In contrast, lncRNAs regulate gene expression in a locus and allele-specific way that is cell-type specific (Parasramka et al., 2016). The specificity of lncRNAs to target different epigenetic modulators at different developmental phases may be a resourceful therapeuticapproach to explore. Furthermore, the current epigenetic drug mechanisms aim to target the overexpressed aberrations of epigenetic enzymes. Because of their role to recruit and activate epigenetic enzymes, lncRNAs may possess therapeutic effects to upregulate poorly expressed epigenetic enzymes. Finally, an individual lncRNA may target only one or a small group of related proteins (Khorkova et al., 2015). Thus this makes them an exceptional approach to avert redundant gene activation.

During muscular diseases including neurogenic atrophy, ALS, spinal muscular atrophy and any loss of nerve supply, MyoG is usually upregulated to promote proteolyisis (Moresi et al., 2010; Bricceno et al., 2012; Galbiati et al., 2012; Mielcarek et al., 2015). Studies found that inhibiting Myparr in denervated muscles resulted in decreased expression of MyoG and increased muscle weight (Hitachi et al., 2019). Hence, Myparr and many other lncRNAs astherapeutic targets in skeletal muscle differentiation may further be an effective novel therapy in muscular disorders.

### Future

Understanding the mechanisms that control and regulate muscle cell development and differentiation enables the research community to improve and enhance the effectiveness of satellite stem cell therapy in muscular disorders. In this review, we outlined the mechanisms of muscle development under the epigenetic control of histone modification and lncRNAs. MRFs reveal to be an excellent factors target to enhance stem cell differentiation into matured muscle fibre for muscle regeneration. Therefore, modulating MRFs via epigenetic mechanisms is showing to be a promising therapeutic approach for muscle differentiation. Histone methylation plays a vital role in modifying the chromatin to switch target genes on or off. Thus, it is a major determining factor for the expression state of genes. Further, emerging studies into lncRNAs provide cogent evidence of their regulatory roles in significant muscle-related signalling pathways. LncRNAs further have an advantageous property of their ability to control myogenesis at specific phases. Hence, they have a high specificity to relevant MRFs. Epigenetic factors do not work in isolation, and lncRNAs have been shown to have significant cross-talks with DNA methyltransferases and histone modifiers. While much progress has been made to explore many of these epigenetic factors during myogenesis, there are still limitations to employ them in clinical use. Firstly, the regulation of histone methylation and lncRNA during myogenesis have only been studied in isolation. Further *in vivo* and *in vitro* investigations are needed for the interplay between lncRNAs modulation with histone modifiers to translate to clinical use. Secondly, harnessing lncRNAs for therapeutic use is still an area that requires much exploration in order for effective clinical use. Finally, augmenting the current trial therapies of HDACi with lncRNAs and histone methylation-mimicking drugs may be a potential therapeutic approach to enhance satellite stem cell implantation and decrease the side effects. Further exploration to advance the knowledge of epigenetic modulation during stem cell activation and muscle development will ultimately progress the regenerative field of muscle cell diseases and muscle cell injury.
